# Machine learning to predict no reflow and in-hospital mortality in patients with ST-segment elevation myocardial infarction that underwent primary percutaneous coronary intervention

**DOI:** 10.1186/s12911-022-01853-2

**Published:** 2022-04-24

**Authors:** Lianxiang Deng, Xianming Zhao, Xiaolin Su, Mei Zhou, Daizheng Huang, Xiaocong Zeng

**Affiliations:** 1 Department of Cardiology, The First Affiliated Hospital of Guangxi Medical University, 6 Shuangyong Road, Nanning, 530021 Guangxi China; 2 Department of Cardiology, The Second People’s Hospital of Nanning, Guangxi, China; 3 Department of Cardiology, The First People’s Hospital of Nanning, Guangxi, China; 4 Department of Cardiology, Guangxi Zhuang Autonomous Region People’s Hospital, Nanning, Guangxi China; 5 Guangxi Key Laboratory Base of Precision Medicine in Cardio-Cerebrovascular Diseases Control and Prevention and Guangxi Clinical Research Center for Cardio-Cerebrovascular Diseases, Nanning, Guangxi China; 6 School of Basic Medical Sciences, Guangxi Medical University, 22 Shuangyong Road, Nanning, Guangxi China

**Keywords:** Machine learning, ST-elevation myocardial infarction, Primary percutaneous coronary intervention, No-reflow, In-hospital mortality

## Abstract

**Background:**

The machine learning algorithm (MLA) was implemented to establish an optimal model to predict the no reflow (NR) process and in-hospital death that occurred in ST-elevation myocardial infarction (STEMI) patients who underwent primary percutaneous coronary intervention (pPCI).

**Methods:**

The data were obtained retrospectively from 854 STEMI patients who underwent pPCI. MLA was applied to predict the potential NR phenomenon and confirm the in-hospital mortality. A random sampling method was used to split the data into the training (66.7%) and testing (33.3%) sets. The final results were an average of 10 repeated procedures. The area under the curve (AUC) and the associated 95% confidence intervals (CIs) of the receiver operator characteristic were measured.

**Results:**

A random forest algorithm (RAN) had optimal discrimination for the NR phenomenon with an AUC of 0.7891 (95% CI: 0.7093–0.8688) compared with 0.6437 (95% CI: 0.5506–0.7368) for the decision tree (CTREE), 0.7488 (95% CI: 0.6613–0.8363) for the support vector machine (SVM), and 0.681 (95% CI: 0.5767–0.7854) for the neural network algorithm (NNET). The optimal RAN AUC for in-hospital mortality was 0.9273 (95% CI: 0.8819–0.9728), for SVM, 0.8935 (95% CI: 0.826–0.9611); NNET, 0.7756 (95% CI: 0.6559–0.8952); and CTREE, 0.7885 (95% CI: 0.6738–0.9033).

**Conclusions:**

The MLA had a relatively higher performance when evaluating the NR risk and in-hospital mortality in patients with STEMI who underwent pPCI and could be utilized in clinical decision making.

## Background

The progressively developing reperfusion strategy has shown significant improvement in the prognosis for patients with ST-elevation myocardial infarction (STEMI) [1]. Since the rapid development of chest pain centers (CPCs) in urban areas in China [2], the primary goal of these centers has been to highlight primary percutaneous coronary intervention (pPCI) to achieve a major reperfusion therapy for patients with evolving STEMI [3]. When a pPCI procedure is carried out, the risk of occurrence of no-reflow (NR) needs to be recognized and managed to improve patient outcomes. NR is defined as inadequate coronary perfusion within the myocardium even after the occluded blood vessels have been opened [4]. It is an important causative factor in the short-term death of STEMI patients who underwent pPCI. The occurrence rate of NR in such cases is approximately 11%–41%, and the subsequent mortality rate is about 7.4%–30.3% [5]. NR is considered a powerful independent predictive factor for in-hospital mortality [6]. When NR occurs, pharmacological therapy becomes difficult as the drugs applied do not reach the distal micro-vessels. To restore normal flow, preventive measures should be taken [7]. Therefore, early prediction, prevention, and proper drug pretreatment are essential in NR treatment [8].

Early risk management and stratification that are pivotal aspects in STEMI patients for early targeted intervention during hospitalization include effective dual antiplatelet therapy, anti-glycoprotein IIb IIIa, statin, optimal anticoagulation, intracoronary calcium inhibitors or adenosine, rotational atherectomy, thrombus aspiration, maintained stable hemodynamically, and intra-aortic balloon pump [1, 8, 9]. Previous studies have documented important risk factors and other predictors, including demographic, clinical, laboratory, and angiographic features. However, previous studies mainly focused on using univariate and multivariate logistic regression analysis to identify the independent variables. They have also detected a lower risk algorithm for NR and in-hospital mortality. This was resolved through machine learning, or artificial intelligence, an emerging field in computer science. Machine learning builds automated data-driven predictive models to program complex problems that include many factors through statistical tools. [10, 11]. A drawback of machine learning is overfitting; however, it can incorporate more factors and analyze complex mathematical models against the traditional statistical models. This study integrated demographic data, clinical characteristics, laboratory parameters, electrocardiogram, echocardiogram, and angiography results to design a scoring and evaluation system using a machine learning approach. This strategy provided a more convenient, practical, and accurate methodology to evaluate and predict NR incidences and in-hospital mortality. With accurate prediction, appropriate interventions can be provided to individuals at the highest risk and thus help reduce the mortality rate. This study focuses on using a multicenter dataset of STEMI patients who underwent pPCI and the feasibility and accuracy of machine learning to predict the incidence of NR and in-hospital mortality to improve medical prospects and decision making. To the best of the authors’ knowledge, this study is the first to evaluate machine learning models on the NR phenomenon, a life-threatening challenge, including demographic, clinical, laboratory, and angiographic features.

## Methods

### Patients

In this retrospective study, data were obtained from STEMI patients who underwent pPCI and were hospitalized at the four National Chest Pain Center Alliance units in Nanning, Guangxi, China (The First Affiliated Hospital of Guangxi Medical University, Guangxi Zhuang Autonomous Region People's Hospital, The First People's Hospital of Nanning, The Second People's Hospital of Nanning) from August 2015 to December 2019. Ethical approval was granted by the Ethics Committee of The First Affiliated Hospital of Guangxi Medical University2021 (KY-E-299). The study was consistent with internationally accepted ethical standards; all methods were carried out according to relevant guidelines and regulations. The inclusion criteria were: (1) persistent typical chest pain > 30 min, with ST-segment elevation in ≥ 2contiguous leads (≥ 0.2mv in precordial leads, ≥ 0.1mv in limb leads), or new left bundle branch block, and an increased level of myocardial injury markers; (2) patients that presented with STEMI within 12 h of the onset of the symptoms and were treated with pPCI; and (3) pPCI strategy could be provided to patients that suffered from chest pain for > 12 h with the presence of any of the following: (1) electrocardiogram (ECG) suggested ongoing ischemia; (2) sustained or recurrent chest pain with dynamic changes in ST-segment; and (3) sustained or recurrent chest pain, with a composite of malignant arrhythmias, heart failure, or shock [1]. The exclusion criteria were: (1) patients having hypersensitivity to antiplatelet agents, anticoagulation drugs, or iodinated contrast; (2) patients showing contraindications to anticoagulant treatment; (3) patients with a previous history of coronary artery bypass surgery; (4) patients with severe hepatic and kidney disorders; (5) patients with autoimmune diseases or malignant tumors; and (6) patients with recent severe infections. The primary outcome of this study was the analysis of the NR and in-hospital mortality rate, which could be identified from the angiography results, PCI procedure reports, and discharge status. NR was defined as post-PCI thrombolysis in a myocardial infarction trial (thrombolysis and thrombin inhibition in myocardial infarction (TIMI)) flow grade that showed ≤ 2 in the infarction related artery, with the exclusion of obstruction due to dissection, spasm, apparent thrombus, or residual stenosis [12, 13].

### Feature selection

An extensive literature search was conducted to determine the potential factors influencing NR and in-hospital mortality. The most eligible features were extracted and noted according to previous literature reports. Then, all the potentially eligible features were selected after discussions with cardiologists and statisticians. The variables included in this study were demographic data, clinical characteristics, laboratory parameters, electrocardiogram, echocardiogram, and angiography results. Body mass index (BMI) is one of the dependent variables of NR and in-hospital mortality, which was excluded from the models for the missing values that were > 5%. The study of NR provided a complete set of predictors for 36 variables (21 continuous variables, 15 categorical variables) (Table 1). The study of in-hospital mortality provided a complete set of predictors (the occurrence rate of NR was regarded as a variable) of 37 variables (21 continuous variables, 16 categorical variables) (Table 2). In the baseline data analysis, a univariate analysis was performed by SPSS (SPSS version 22.0; SPSS Chicago, IL, USA) (*p* < 0.05 was considered statistically significant).

**Table 1:** Baseline characteristics of the NR study population (*n* = 854)

Variable	Normal blood flow (*n* = 755)	NR (*n* = 99)	*p-*value
Age (years)	60.34 (13.27)	62.79 (13.88)	0.086
Male (*n* (%))	628 (83.2)	74 (74.7)	0.039
Hypertension (*n* (%))	345 (45.7)	38 (38.4)	0.169
Diabetes (*n* (%))	124 (16.4)	12 (12.1)	0.271
Smoke (*n* (%))	377 (49.9)	34 (34.3)	0.004
Angina pectoris (*n* (%))	41 (5.4)	7 (7.1)	0.505
Previous PCI (*n* (%))	19 (2.5)	5 (5.1)	0.267
Anterior wall MI (*n*(%))	391 (51.8)	48 (48.5)	0.536
Cardiac troponin I (normalized)	0.47 (0.33)	0.67 (0.31)	< 0.001
creatine kinase-MB (normalized)	0.13 (0.13)	0.22 (0.17)	< 0.001
hs-CRP (normalized)	0.34 (0.33)	0.50 (0.39)	< 0.001
Hemoglobin (g/L)	137.65 (19.28)	133.90 (19.60)	0.07
WBC count (10^9^/L)	11.50 (3.94)	12.40 (3.78)	0.033
Creatinine (μmol/L)	89.68 (43.15)	99.38 (45.22)	0.037
Uric acid (μmol/L)	385.33 (118.53)	407.30 (135.37)	0.089
Albumin (g/L)	38.72 (4.37)	38.43 (4.91)	0.544
Glucose (mmol/L)	7.31 (3.44)	7.52 (2.84)	0.577
LDL-C (mmol/L)	3.28 (1.05)	3.12 (0.97)	0.156
HDL-C (mmol/L)	1.20 (0.44)	1.15 (0.32)	0.351
Triglyceride (mmol/L)	1.65 (1.13)	1.60 (1.25)	0.734
Total cholesterol (mmol/L)	5.01 (1.24)	4.89 (1.26)	0.347
Fibrinogen (g/L)	3.65 (1.08)	3.63 (1.24)	0.862
Shock (%)	13 (1.7)	7 (7.1)	0.003
Malignant arrhythmia (*n* (%))	90 (11.9)	18 (18.2)	0.078
**Killip class (*n* (%))**			
I	610 (80.8)	64 (64.6)	< 0.001
II	83 (11.0)	15 (15.2)	0.222
III	15 (2.0)	3 (3.0)	0.758
IV	47 (6.2)	17 (17.2)	< 0.001
LVEF (%)	57.97 (9.15)	54.30 (10.16)	< 0.001
Total ischemia time (h)	4.85 (3.51)	6.29 (5.61)	< 0.001
SO-to-FMC (min)	157.21 (166.20)	262.06 (290.03)	< 0.001
Door-to balloon (min)	85.68 (49.34)	88.53 (54.05)	0.594
**Baseline TIMI flow**			
0	581 (77.0)	90 (90.9)	0.001
1	38 (5.0)	5 (5.1)	1.0
2	77 (10.2)	1 (1.0)	0.003
3	59 (7.8)	3 (3.0)	0.085
**Criminal blood vessels (*n* (%))**			
LAD	431 (57.3)	49 (49.5)	0.140
LCX	56(7.4)	8 (8.1)	0.814
RCA	263 (34.8)	41 (41.4)	0.199
LM	5 (0.7)	1 (1.0)	0.524
Double or multiple lesions (*n* (%))	501 (66.4)	78 (78.8)	0.013
Post dilatation (n (%))	446 (59.1)	56 (56.6)	0.634
Thrombus aspiration (*n* (%))	84 (11.1)	29 (29.3)	< 0.001
Stent diameter (mm)	3.14 (0.43)	3.14 (0.40)	0.895
Stent length (mm)	33.94 (17.25)	41.57 (18.78)	< 0.001

*hs-CRP* high-sensitivity C-reactive protein, *HDL-C* high-density lipoprotein cholesterol, *LVEF* left ventricular ejection fraction, *LAD* left anterior descending coronary artery, *LCX* left coronary circumflexus artery, *LDL-C* low-density lipoprotein cholesterol, *LM* left main coronary artery, *MI* myocardial infarction, *PCI* percutaneous transluminal coronary intervention, *RCA* right coronary artery, *SO-to-FMC* symptom-onset to-first medical contact, *TIMI* thrombolysis and thrombin inhibition in myocardial infarction

**Table 2:** Baseline characteristics of in-hospital mortality study population (*n* = 854)

Variable	Better discharge (*n* = 807)	In-hospital mortality (*n* = 47)	*p-*value
Age (years)	60.09 (13.20)	69.83 (12.72)	< 0.001
Male (*n* (%))	674 (83.5)	28 (59.6)	< 0.001
Hypertension (*n* (%))	362 (44.9)	21 (44.7)	0.981
Diabetes (*n* (%))	122 (15.1)	14 (29.8)	0.008
Smoke (*n* (%))	394 (48.8)	17 (36.2)	0.091
Angina pectoris (*n* (%))	43 (5.3)	5 (10.6)	0.124
Previous PCI (*n* (%))	21 (2.6)	3 (6.4)	0.284
Anterior wall MI (*n* (%))	412(51.1)	27(57.4)	0.394
Cardiac troponin I (normalized)	0.47 (0.33)	0.76 (0.32)	< 0.001
Creatine kinase-MB (normalized)	0.14 (0.13)	0.30 (0.24)	< 0.001
hs-CRP (normalized)	0.33 (0.33)	0.73 (0.35)	< 0.001
Hemoglobin (g/L)	137.73 (19.16)	128.38 (20.53)	0.001
WBC count (10^9^/L)	11.47 (3.82)	13.95 (5.00)	< 0.001
Creatinine (μmol/L)	89.20 (42.11)	118.37 (56.25)	< 0.001
Uric Acid (μmol/L)	382.15 (116.94)	486.27 (141.94)	< 0.001
Albumin (g/L)	38.88 (4.28)	35.30 (5.50)	< 0.001
Glucose (mmol/L)	7.26 (3.35)	8.59 (3.68)	0.009
LDL-C (mmol/L)	3.27 (1.04)	3.18 (1.12)	0.59
HDL-C (mmol/L)	1.19 (0.43)	1.18 (0.33)	0.92
Triglyceride (mmol/L)	1.66 (1.17)	1.38 (0.63)	0.111
Total cholesterol (mmol/L)	5.01 (1.23)	4.84 (1.44)	0.372
Fibrinogen (g/L)	3.63 (1.07)	3.94 (1.55)	0.061
Shock (*n* (%))	13 (1.6)	7 (14.9)	< 0.001
Malignant arrhythmia (n (%))	95 (11.8)	13 (27.7)	0.001
**Killip class (*n* (%))**			
I	663 (82.2)	11 (23.4)	< 0.001
II	89 (11.0)	9 (19.1)	0.090
III	14 (1.7)	4 (8.5)	0.014
IV	41 (5.1)	23 (48.9)	< 0.001
LVEF (%)	58.05 (9.02)	48.88 (10.50)	< 0.001
Total ischemia time (h)	4.94 (3.76)	6.42 (4.81)	0.01
SO-to-FMC (m)	163.53 (178.73)	269.81 (287.02)	< 0.001
Door-to-balloon (m)	85.49 (50.01)	94.98 (47.24)	0.205
**Baseline TIMI flow (*n* (%))**			
0	628 (77.8)	43 (91.5)	0.026
1	40 (5.0)	3 (6.4)	0.927
2	78 (9.7)	0 (0.0)	0.017
3	61 (7.6)	1 (2.1)	0.269
**Criminal blood vessels (*n* (%))**			
LAD	455(56.4)	25 (53.2)	0.668
LCX	60 (7.4)	4 (8.5)	1.0
RCA	290 (35.9)	14 (29.8)	0.392
LM	2 (0.2)	4 (8.5)	< 0.001
Double or multiple lesions (*n* (%))	538 (66.7)	41 (85.4)	0.007
Post dilatation (*n* (%))	480 (59.5)	22 (46.8)	0.086
Thrombus aspiration (*n* (%))	104 (12.9)	9 (19.1)	0.218
Stent diameter (mm)	3.14 (0.43)	3.03 (0.36)	0.066
Stent length (mm)	34.39 (17.30)	42.29 (20.86)	0.003
NR (*n* (%))	83(10.3)	16(34.0)	< 0.001

*hs-CRP* high-sensitivity C-reactive protein, *HDL-C* high-density lipoprotein cholesterol, *LAD* left anterior descending coronary artery, *LCX* left coronary circumflexus artery, *LDL-C* low-density lipoprotein cholesterol, *LM* left main coronary artery, *LVEF* left ventricular ejection fraction, *MI* myocardial infarction, *PCI* Percutaneous Transluminal Coronary Intervention, *RCA* right coronary artery, *SO-to-FMC* symptom-onset to-first medical contact, *TIMI* thrombolysis and thrombin inhibition in myocardial infarction

### Data processing

Some continuous variables, which included cardiac troponin I (cTnI), creatine kinase-MB (CK-MB), and high-sensitivity C-reactive protein (hs-CRP), were normalized (from 0 to 1) because of inconsistent value ranges and units. For predictors with missing values < 5%, this was filled up with the mean value, a general and widely adopted method used to practice resolving the missing values in machine learning [14]. Before model building, value correlation was carried out to eliminate the variables that might cause numerical instability, which might lead to model overfitting or worsen, or both the interpretability of the model and value correlation of variables. The correlation analyses used Pearson’s correlation coefficient and were performed using Sanger Box software [11].

### Supervised machine learning methods

**Fig. 1:**
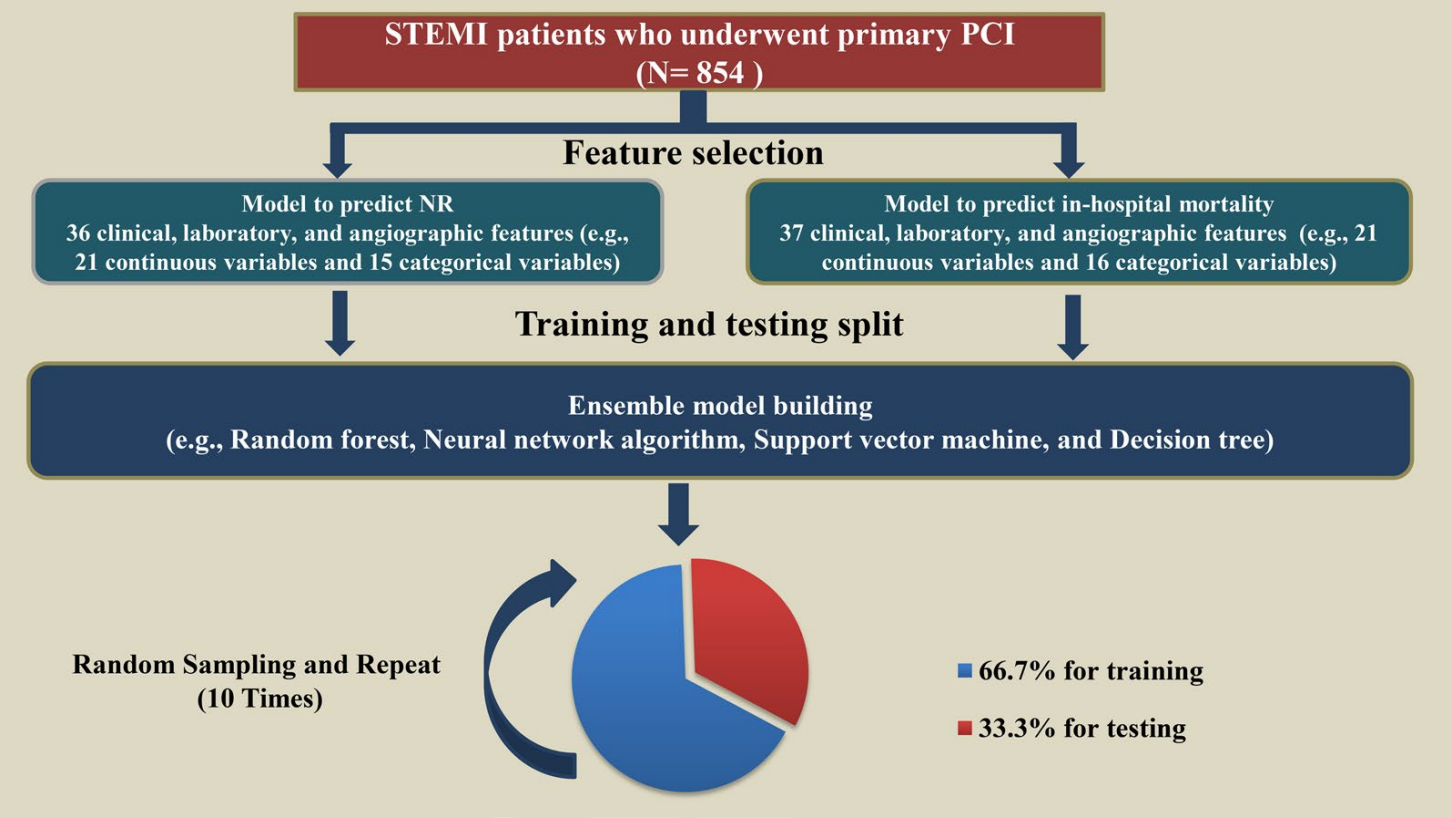
Schematic workflow for AI*.* The AI method to predict NR and in-hospital mortality included feature selection, training, and testing split, which combined four different model algorithms (e.g., RAN, NNET, SVM, and CTREE). Random sampling was conducted 10 times, and the results were averaged. AI, artificial intelligence; CTREE, Decision Tree; NNET, Neural Network Algorithm; RAN, random forest; SVM, Support Vector Machine

The workflow of the supervised machine learning models that used the clinical, laboratory, and angiographic variables is shown in Fig. 1. Based on the data from the training set, four supervised machine learning methods were used to develop the predictive classifiers: (1) random forest (RAN); (2) neural network algorithm (NNET); (3) support vector machine (SVM); and (4) decision tree (CTREE).

(1) RAN: This is an integrated learning algorithm based on a decision tree. It is easy to implement, has advantages over other algorithms in many data sets, and performs well in classification and regression. It can handle very high-dimensional data and does not need to carry out feature selection (because the feature subset is selected at random); after training, RAN provided the significant features. In addition, the ability to resist overfitting is relatively strong. Compared with decision trees, RANs are slower because they are ensemble methods.

The steps to construct the RAN for classification were: (1) there are *N* times of extraction for putting back from a sample of size Q, one at a time, and finally, *N* samples are formed. The *N* samples selected were used to train the decision tree as the samples at the root node of the decision tree. (2) when each sample has *M* attributes, and each decision tree node needs to be split, m attributes are randomly selected from the *M* attributes to meet the condition *m* ≪ *M*. Then, a specific strategy (i.e., information gain) was adopted from the *M* attributes to select one attribute as the splitting attribute of the node. (3) to form a decision tree, each node should split according to step 2 until it can no longer split; and (4) many decision trees are established according to steps 1–3, which constitutes a random forest.

(2) NNET: This realizes a nonlinear mapping function from input to output, automatically extracts the reasonable rules between the input and output data through learning, and adaptively memorizes the learning content in the weight of the network. It has a strong generalization and fault tolerance. However, the network is overly sensitive compared with the initial network weights. Initializing the network with different weights tended to converge to different local minima; therefore, each training could obtain different results. The convergence speed of the algorithm is slow, and there is no unified and complete theoretical guidance for the selection of the network structure. In general, it can only be selected by experience;

The steps to construct the NNET for classification were: (1) obtain the input of the neural network. This extracts the feature vector in the problem entity as the input of the neural network; (2) defines the structure of the neural network and how to get the output from the input in the neural network. This process is the forward propagation algorithm of the neural network: (3) train the neural network. The neural network parameters, for example, weight and threshold, were obtained from the training data. Network optimization is often required in this process, and the most commonly used method is a backpropagation algorithm. This process needs to define a loss function to describe the gap between the current predicted value and the real data, then adjust the value of neural network parameters through a backpropagation algorithm to narrow the gap: and (4) predict where the trained network is used for data prediction.

(3) SVM uses the integral operator kernel functions to replace the nonlinear mapping to the high-dimensional space. This avoids the traditional process from induction to deduction and achieves efficient training from training samples to forecasting samples. Transduction reasoning significantly simplifies the usual classification and regression problems. However, it is not sensitive to outliers, and it is easy to catch important samples and remove a large number of redundant samples. Therefore, the algorithm is simple and has good robustness. But SVM uses quadratic programming to solve the support vector, and to solve the quadratic programming involves the calculation of the m-order matrix (*m* = number of samples). When m is large, the storage and calculation of the matrix will consume a lot of machines’ memory and computation time. SVM is sensitive to the selection of parameters and kernel functions, and to date, there is not a good method to solve the problem of selecting kernel functions;

The steps to construct the SVM for classification were: (1) prepare the dataset and convert it to the data format supported by SVM; (2) simple scaling of the data; (3) Kernel function selection (i.e., radial function); (4) cross-validation (i.e., using fivefold cross-validation) and select the best parameters c and g; (5) SVM model is obtained by training the whole training set with the obtained optimal parameters c and g; and (6) The obtained SVM model is used for test classification.

The excavated classification rules are highly accurate, easy to understand, generate understandable rules, do not require domain knowledge or parametric assumption, and are suitable for high-dimensional data.

(4) CTREE: The calculation speed of this method is fast, and it is easy to transform into classification rules. The disadvantage is that for data with inconsistent sample sizes in each category, the information gained is biased toward those with more values. It is easy to overfit, which ignores the correlation between attributes.

The starting point to choose RANs and CTREEs is to compare the classification of single trees and ensemble trees. SVMs and NNET implement nonlinear mapping. In addition, SVMs, NNET, and RANs can be regarded as black-box models. Therefore, the three methods are compared and studied.

The steps to construct the CTREE for classification were: (1) initialize feature set and data set; (2) information entropy of the data set and the conditional entropy of all features were calculated, and the feature with the largest information gain was selected as the current decision node; (3) update the data set and feature set (e.g., delete the feature used in the previous step, and divide the data set for different branches according to the feature value); and (4) repeat steps 2 and 3. If the subset value contains a single feature, it is a branch leaf node.

All machine learning operations were performed using the R language platform by calling the program package.

The parameter settings were: (1) RAN or random forest function in the random forest package, which provided the function for using the random forest algorithm to solve classification and regression problems. Here, the application of random forest algorithms for classification problems was focused on. The random forest function was used for model training. One of the necessary parameters of the random forest algorithm is the number of features that can be searched at each segmentation point. A good default value for classification is *M* = sqrt (*P*), where *M* is the number of features that can be searched at the segmentation point and *P* is the total number of input variables. The number of trees is obtained when the error of data outside the bag is the smallest. The others were default values; (2) the R language platform provided an algorithm package NNET that dealt with the artificial neural network. This algorithm provided the implementation of the traditional feed-forward and backpropagation neural network algorithm. The hidden node (n2) and the input node (n1) in the three-layer NNET were related by n2 = 2n1 + 1, where *M* is the number of features. The weight adjustment speed was 0.1, and the maximum number of iterations was 1,000; (3) the e1071 software package was used to complete the data analysis and mining based on SVM. One of the most important functions in this package is the SVM function that can build the SVM model. The SVM can be used as a classification machine, regression machine, or novelty detection.

Depending on whether the output is a factor or not, the default setting for type is C-classification or eps-regression, respectively, but might be overwritten by setting an explicit value. In this paper, the method was C-classification, kernel as radial (the kernel used in training and predicting), because SVM is a nonlinear model when adjusting the values of parameters c (cost of constraints violation) and g (parameter needed for all kernels except linear), an initial value will be given, and then multiply it by 0.1 or 10 as a step according to the performance of the model. When the approximate range is determined, refine the search interval. c as 10 and g was 0.1, obtained in the end; (4) use the rpart package with the ID3 algorithm. The processing for rpart was: independent variables, and all segmentation points were evaluated first. The best choice of the evaluation was to make the data in the segmented group pure, where pure means that the variation in the value of the dependent variable of the data in the group was small. The rpart package's default measure for pure was the Gini value. Many parameters determine the stop division. It is very important to determine these parameters because for a finer division, the more complex the model, which is more prone to overfitting, and the coarser the division, the lower the fitting. This problem is usually handled using the pruning method. First, establish a tree model with a fine and complex division, then estimate the error of each model under different pruning conditions according to the cross-validation method and select the tree model with the smallest error. In this paper, the method took class, and the rest took default values.

To evaluate the efficacy of each model, a random sampling method was applied to split the entire data into training (66.7%) and testing (33.3%) sets. Random sampling was conducted 10 times, and the final value was the average value of the 10 operation results. The AUC and 95% CI were calculated, and the ROC curves were drawn. There was limited data on in-hospital deaths (47/854) or NR (99/854) in the samples, which meant that the classification evaluation index, such as the F1 score, was difficult to obtain because the F1 score would be zero. A variable importance plot was produced after performing the training with the training data set (66.7%) by using the importance function in the random forest model. The programming language adopted the R version 4.05.

## Results

### Patient characteristics

Between August 2015 and December 2019, 907 consecutive STEMI patients were screened who underwent pPCI in four CPCs. Among them, 2 patients had a previous history of coronary artery bypass surgery, 10 had severe hepatic and kidney disorders, 1 had autoimmune diseases, 8 had malignant tumors, 5 had recent severe infections, and 27 had pPCI procedures within an unreasonable time frame were excluded. Following the exclusions, 854 patients were included in the subsequent analyses. The patients were categorized into a normal blood flow (*n* = 775) and the NR (*n* = 99) group. Their demographic, clinical, laboratory and angiographic parameters are summarized in Table 1. The included 854 patients were further categorized into discharged (*n* = 807) and in-hospital mortality (*n* = 47) groups, and their demographic, laboratory, clinical, and angiographic parameters are summarized in Table 2.

### Variables examined

**Fig. 2:**
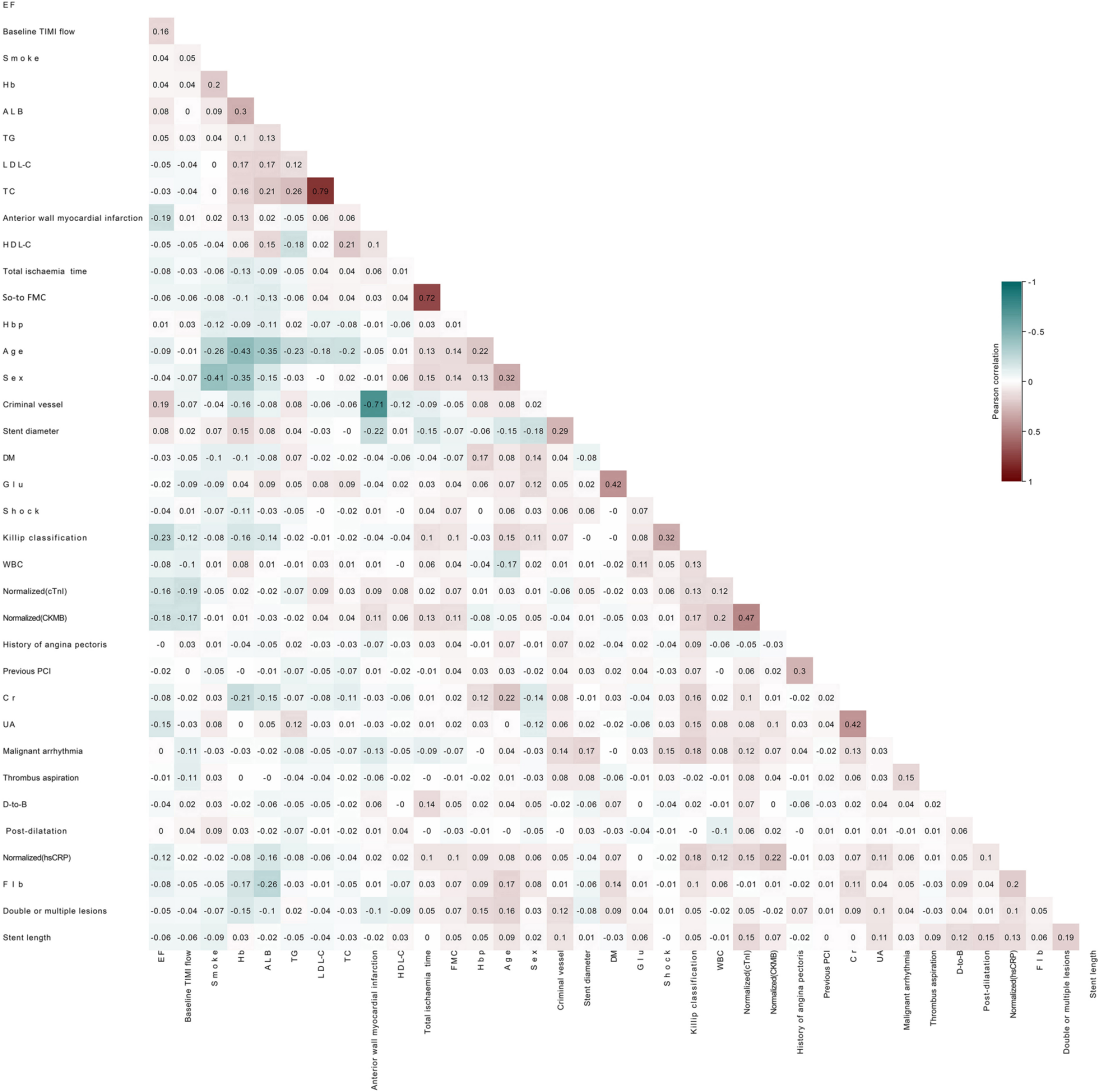
Correlation analysis of the variables in the dataset to predict NR, as shown in the heat map, the dataset was not composed of many correlated variables, which indicated the model was simpler and more stable. ALB, albumin; Cr, creatinine; CK-MB, creatine kinase-MB; cTnI, cardiac troponin I; DM, diabetes mellitus; D-to-B, door-to-balloon; EF, ejection fraction; Fib, Fibrinogen; Glu, glucose; Hb, hemoglobin; Hbp, high blood pressure; HDL-C, high-density lipoprotein cholesterol; hs-CRP, high-sensitivity C-reactive protein; LDL-C, low-density lipoprotein cholesterol; PCI, percutaneous coronary intervention; SO-to-FMC, symptom-onset to-first medical contact; TC, total cholesterol; TG, triglyceride; TIMI, thrombolysis and thrombin inhibition in myocardial infarction; UA, uric acid; WBC, white blood cells

**Fig. 3:**
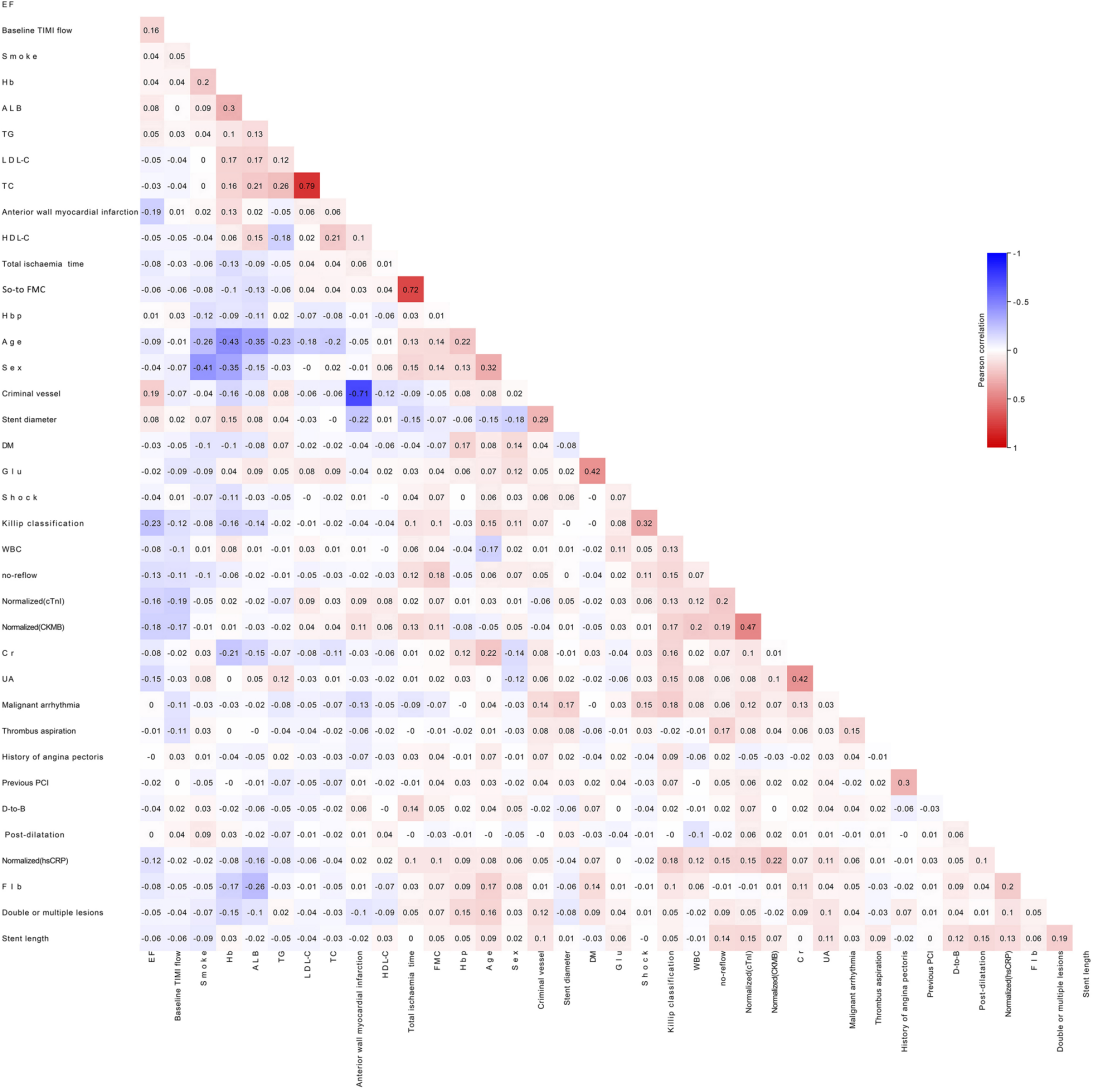
Correlation analysis of the variables in the dataset for the prediction of in-hospital mortality, the dataset was not composed of many correlated variables, which indicated the model was simpler and more stable. ALB, albumin; Cr, creatinine; CK-MB, creatine kinase-MB; cTnI, cardiac troponin I; DM, diabetes mellitus; D-to-B, door-to-balloon; EF, ejection fraction; Fib, Fibrinogen; Glu, glucose; Hb, hemoglobin; Hbp, high blood pressure; HDL-C, high-density lipoprotein cholesterol; hs-CRP, high-sensitivity C-reactive protein; LDL-C, low-density lipoprotein cholesterol; PCI, percutaneous coronary intervention; SO-to-FMC, symptom-onset to-first medical contact; TC, total cholesterol; TG, triglyceride; TIMI, thrombolysis and thrombin inhibition in myocardial infarction; UA, uric acid; WBC, white blood cells

The value correlation graph shows that the data set did not contain many relevant variables for the NR study (Fig. 2) and in-hospital mortality study (Fig. 3). Therefore, none of the other independent variables were excluded based on the correlation coefficient and clinical significance.

### NR and in-hospital mortality prediction

**Fig. 4:**
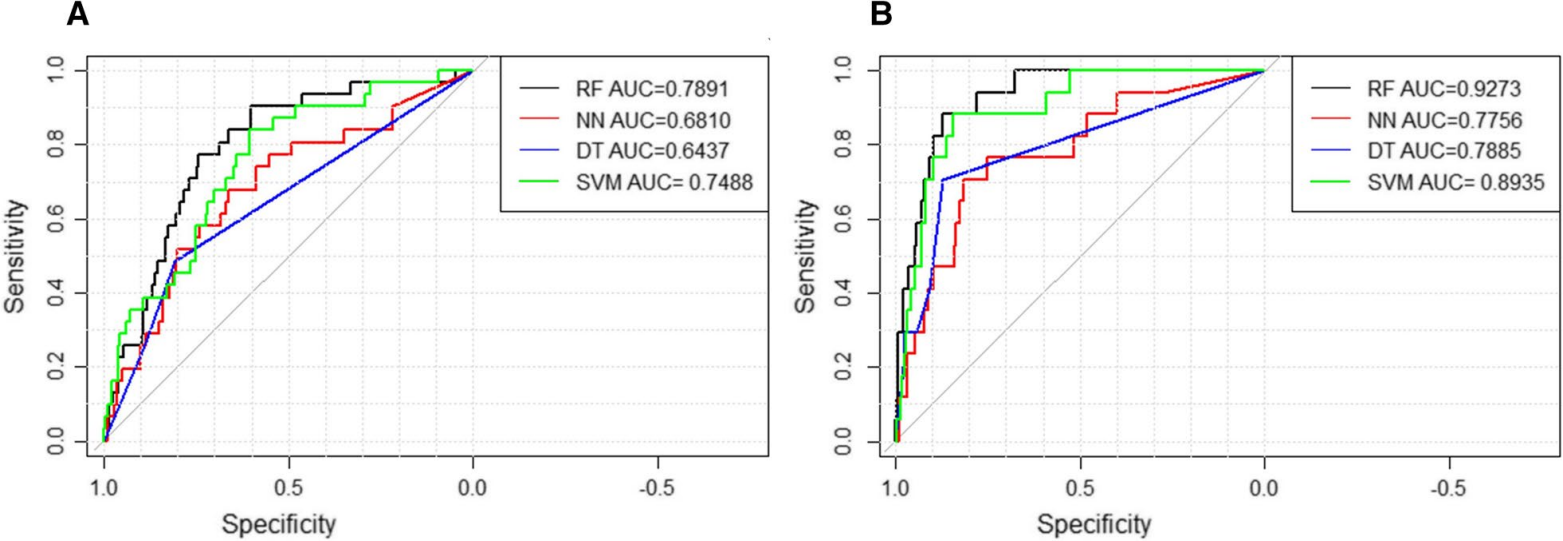
ROC curves to predict NR and in-hospital mortality: **A** a RAN model presented a higher AUC for NR; and **B** in-hospital mortality prediction than all other models (e.g., NNET, SVM, and CTREE). AUC, area under the curve; CTREE, Decision Tree; NNET, Neural Network Algorithm; RAN, random forest; SVM, Support Vector Machine

An optimal AUC (0.7891, 95% CI: 0.7093–0.8688) for NR was derived from the RAN model, and the AUC of the SVM model was 0.7488 (95% CI: 0.6613–0.8363), the AUC of NNET was 0.681 (95% CI: 0.5767–0.7854), and the AUC of the CTREE was 0.6437 (95% CI:0.5506–0.7368) (Fig. 4A).

An optimal AUC (0.9273, 95% CI: 0.8819–0.9728) for in-hospital mortality was derived from the RAN model, and the AUC of the SVM model was 0.8935 (95% CI:0.826–0.9611), the AUC of the NNET was 0.7756 (95% CI: 0.6559–0.8952), and the AUC of the CTREE was 0.7885 (95% CI:0.6738–0.9033) (Fig. 4B).

### Feature importance ranking

**Fig. 5:**
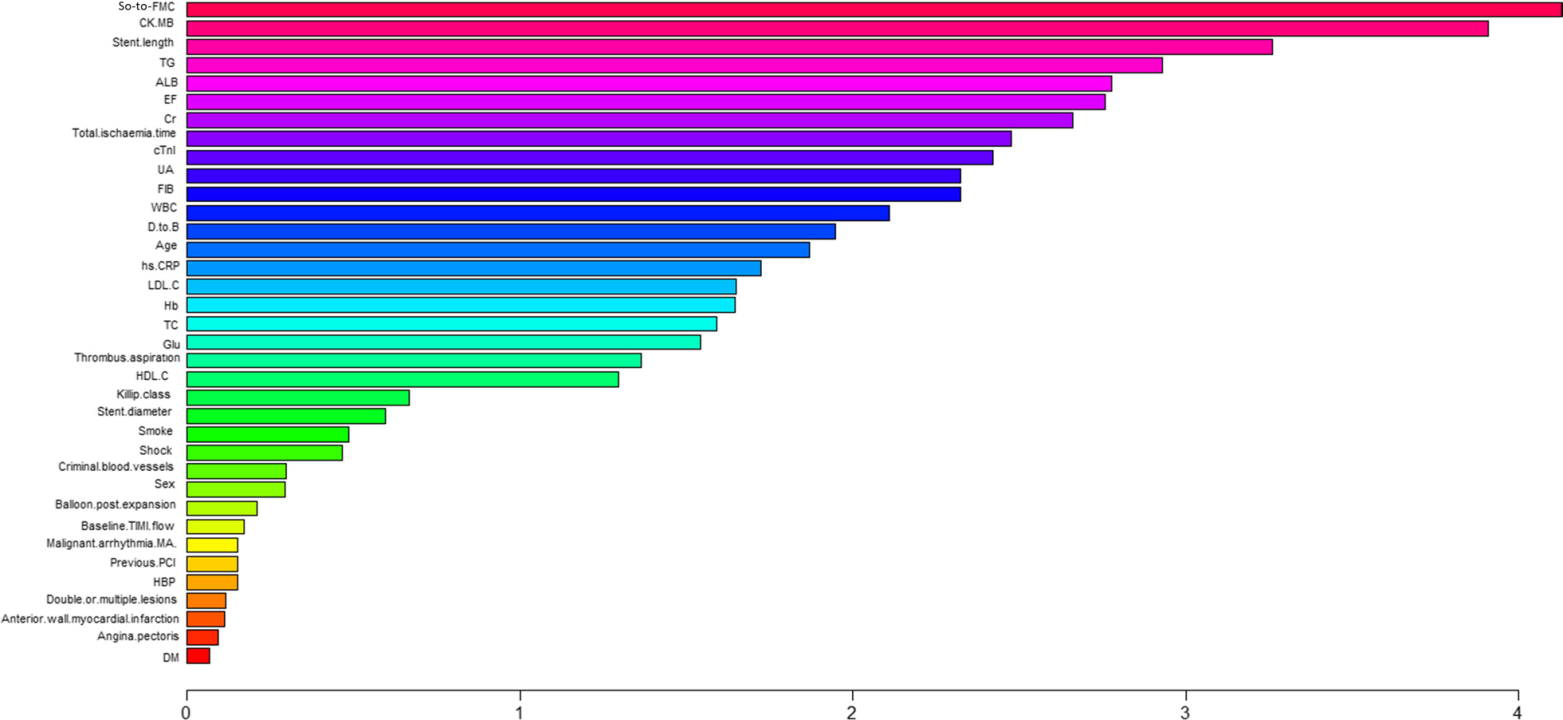
Ranking of feature importance to predict NR: Contribution of clinical, laboratory, and angiographic variables to the optimal AI-based prediction model (RAN). ALB, albumin; Cr, creatinine; CK-MB, creatine kinase-MB; cTnI, cardiac troponin I; DM, diabetes mellitus; D-to-B, door-to-balloon; EF, ejection fraction; FIB, Fibrinogen; Glu, glucose; Hb, hemoglobin; HBP, high blood pressure; HDL-C, high-density lipoprotein cholesterol; hs-CRP, high-sensitivity C-reactive protein; LDL-C, low-density lipoprotein cholesterol; MA, Malignant arrhythmia; PCI, percutaneous coronary intervention; RAN, random forest; SO-to-FMC, symptom-onset to-first medical contact; TC, total cholesterol; TG, triglyceride; TIMI, thrombolysis and thrombin inhibition in myocardial infarction; UA, uric acid; WBC, white blood cells

**Fig. 6:**
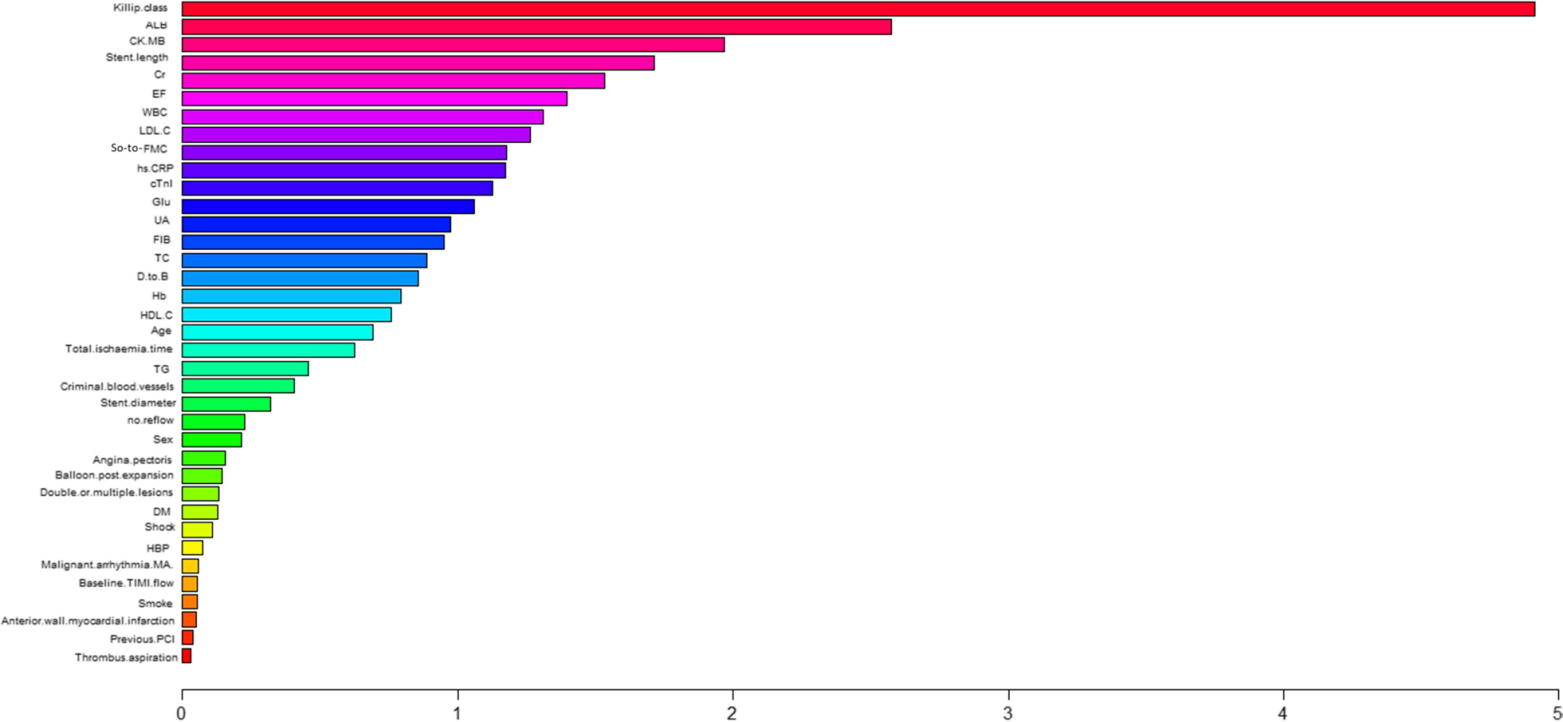
Ranking of feature importance to predict in-hospital mortality: Contribution of clinical, laboratory, and angiographic variables to the optimal AI-based prediction model (RAN). ALB, albumin; Cr, creatinine; CK-MB, creatine kinase-MB; cTnI, cardiac troponin I; DM, diabetes mellitus; D-to-B, door-to-balloon; EF, ejection fraction; FIB, Fibrinogen; Glu, glucose; Hb, hemoglobin; HBP, high blood pressure; HDL-C, high-density lipoprotein cholesterol; hs-CRP, high-sensitivity C-reactive protein; LDL-C, low-density lipoprotein cholesterol; MA, Malignant arrhythmia; PCI, percutaneous coronary intervention; RAN, random forest; SO-to-FMC, symptom-onset to-first medical contact; TC, total cholesterol; TG, triglyceride; TIMI, thrombolysis and thrombin inhibition in myocardial infarction; UA, uric acid; WBC, white blood cells

Figures 5 and 6 depict the relative importance of the variables when predicting NR and in-hospital mortality that used an optimal machine, learning-based model. In the RAN model, SO-to-FMC, CK-MB, stent length, triglyceride (TG), Alb, LVEF, Cr, cTnI, and total ischemia time were relatively important independent variables to predict NR (Fig. 5); however, Killip class, Alb, CK-MB, stent length, Cr, LVEF, WBC, low-density lipoprotein cholesterol (LDL-C), SO-to-FMC, hs-CRP, and cTnI were relatively important independent variables for in-hospital mortality prediction (Fig. 6).

### Model validation between different centers

**Fig. 7:**
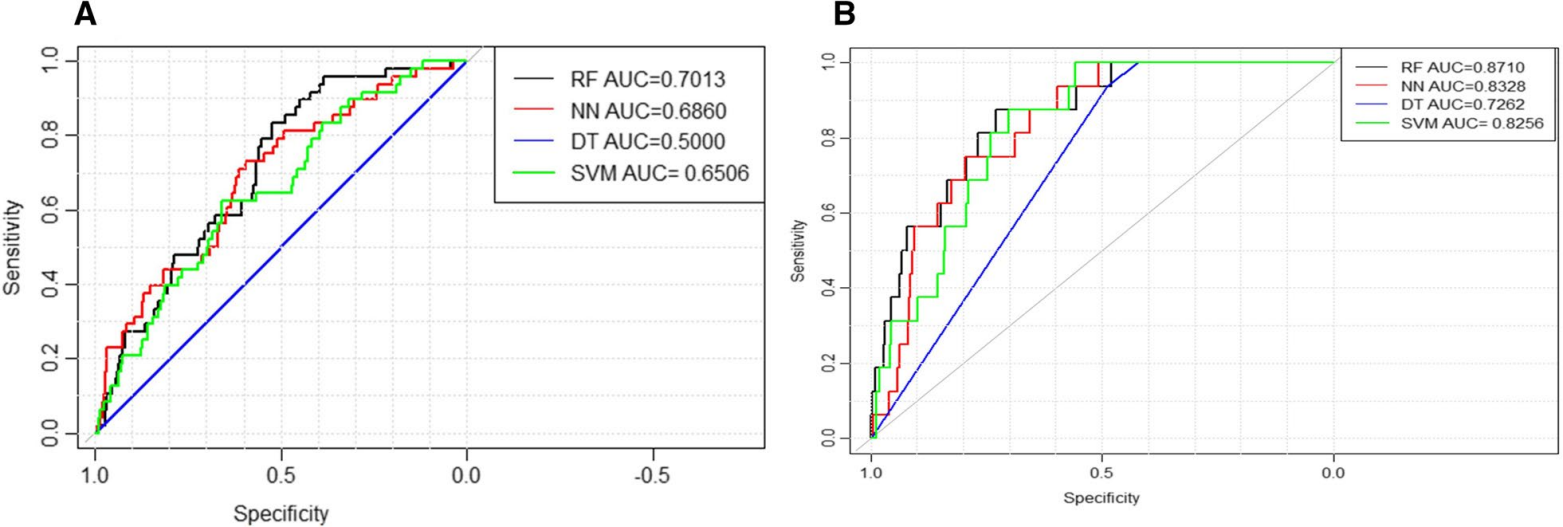
Model validation between different centers, one center (The Second People's Hospital of Nanning) as a training set and another center (The First People's Hospital of Nanning) as a testing set, ROC curves to predict NR and in-hospital mortality: **A** a RAN model presented a higher AUC for NR; and **B** in-hospital mortality prediction than all other models (NNET, SVM, and CTREE). AUC, area under the curve; CTREE, Decision Tree; NNET, Neural Network Algorithm; RAN, random forest; SVM, Support Vector Machine

To validate the model, we attempted to analyze the effect of the training models on the data from one center and tested it on another. Two centers were randomly selected from the four used in this study and matched in different ways, using one center (The Second People's Hospital of Nanning) as a training set and another center (The First People's Hospital of Nanning) as testing was better. An optimal AUC (0.7013, 95% CI: 0.6318–0.7709) for NR was derived from the RAN model, and the AUC of the SVM model was 0.6506 (95% CI: 0.5712–0.7301), the AUC of NNET was 0.6860 (95% CI: 0.6065–0.7655), and the AUC of the CTREE was 0.5000 (95% CI: 0.5000–0.5000) (Fig. 7A).

An optimal AUC (0.871, 95% CI: 0.7933–0.9488) for in-hospital mortality was derived from the RAN model, and the AUC of the SVM model was 0.8256 (95% CI: 0.7531–0.8981), the AUC of the NNET was 0.8328 (95% CI: 0.7574–0.9083), and the AUC of the CTREE was 0.7262 (95% CI: 0.6822–0.7702) (Fig. 7B).

### Statistical analysis was performed by controlling demographic factors

**Fig. 8:**
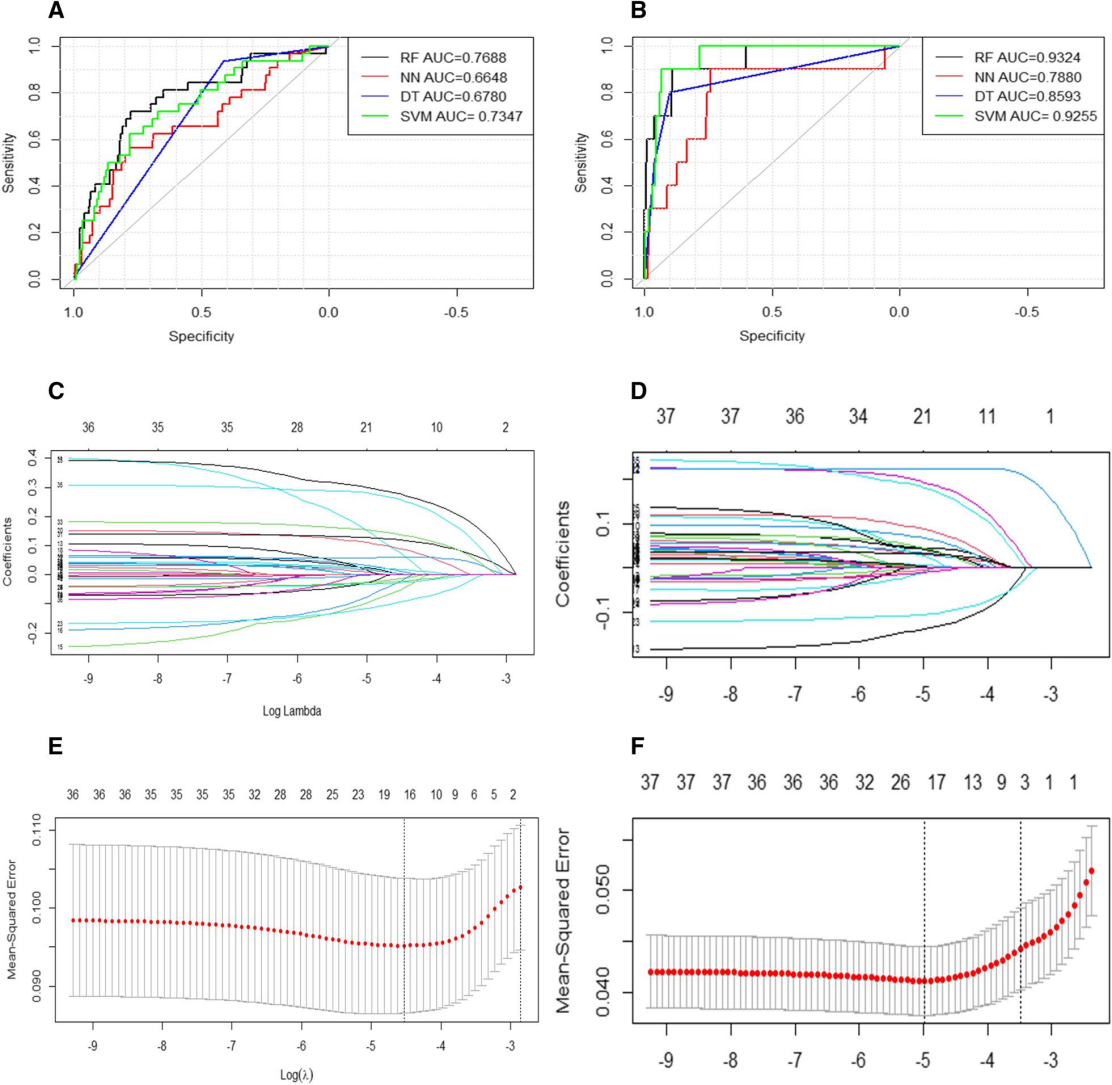
Statistical analysis was performed by controlling demographic factors. Machine learning procedure was performed after removal of age and gender. ROC curves to predict NR and in-hospital mortality: **A** a RAN model presented a higher AUC for NR and; **B** in-hospital mortality prediction than all other models (NNET, SVM, and CTREE). Texture feature selection using the least absolute shrinkage and selection operator (Lasso) binary logistic regression model. The tuning parameter (λ) selection in the Lasso model was based on minimum criteria. **C** Lasso coefficient profiles of the 36 features for NR; **D** Lasso coefficient profiles of the 37 features for in-hospital mortality; coefficient profile plot was produced against the log (λ) sequence; **E** AUC curve was plotted versus log (λ) for NR; and **F** in-hospital mortality. Dotted vertical lines were drawn at the optimal values using the minimum criteria and one standard error of the minimum criteria. AUC, area under the curve; CTREE, Decision Tree; NNET, Neural Network Algorithm; RAN, random forest; SVM, Support Vector Machine

The machine learning procedure was performed with the remaining metrics after removing age and gender to control the influence of the demographic factors. The results showed that the corresponding AUC values (Fig. 8A, B) did not change significantly. Therefore, the Lasso algorithm was performed to screen the variables by calling the R language package glmnet and setting the following parameters: family = "gaussian", nlambda = 100, alpha = 1. To obtain the relationship between the mean square error and the number of variables, the function cv.glmnet was called, and the parameter was set: type. measure = "mse", alpha = 1, family = "gaussian". Following variables screening (Fig. 8C–F), age and gender had little effect on the outcome, especially in-hospital mortality. The direct removal of the demographic factors could be considered.

## Discussion

This pilot study was designed to analyze whether an MLA could be used to benefit STEMI patients. The major implications and findings of this multicenter study were that compared to the other proposed models, the RAN exhibited the highest discriminatory performance for the prediction of NR (AUC, 0.7891) and in-hospital mortality (AUC, 0.9273) in STEMI patients who underwent pPCI.

Various studies have developed predictive models for the occurrence of NR in STEMI patients who underwent pPCI. These models were based on variables such as the atherogenic index of plasma (log TG/HDL-C) [15], the neutrophil-to-lymphocyte ratio [16], the lymphocyte-to-monocyte ratio [17], the fibrinogen-to-albumin ratio [18], R wave peak time [19], a combination of plasma D-Dimer and endothelin-1 levels [20], plasma D-Dimer and pre-infarction angina [21], a combination of pre-infarction angina and mean platelet volume to lymphocyte count ratio [22], the red cell distribution width–platelet ratio (RPR) [23], CHA2DS2-VASc [24] or CHA2DS2-VASc-HSF score [25], and some new risk scoring system [26]. Based on the AUC values, moderate accuracy was observed in this study, which is similar to previous reports. In general, NR is considered a complex pathophysiological process involving multiple risk factors, which might cause difficulty in the prediction [27]. Compared to traditional statistical methods, machine learning offers a new approach to improve risk prediction. Combining machine learning with big data could be helpful to promote the development of prediction models for NR. Although this study is limited by sample size, an approach that uses machine learning models could bring advantages that highlight the novel outcomes. More details are required on the contribution of each feature for model prediction. The RAN model was the optimal MAL among the four AI models used in this study, and important correlated features were derived from this algorithm. Figure 5 shows that SO-to-FMC is the most important factor when predicting NR with the RAN model, followed by CK-MB, stent length, TG, Alb, LVEF, Cr, cTnI, and total ischemia time. In addition, previous studies have demonstrated that delayed reperfusion, decreased EF, elevated CK-MB and cTnI, increased creatinine, and stent length has a larger impact on NR [28]. A recent study confirmed that the low serum albumin level at admission is an independent predictive factor for NR following pPCI in STEMI patients [29]. The coronary slow flow phenomenon is present in patients with lower high-density lipoprotein cholesterol (HDL-C) but higher BMI and TG levels [30]. An independent study confirmed that the atherogenic index of plasma (logTG/HDL-C) is independently associated with NR [15]. These reports support our findings that Alb and TG are important factors when predicting NR in the RAN model.

To date, the TIMI score and Global Registry of Acute Coronary Events (GRACE) score are the most widely applied death risk scores in acute coronary syndromes. A recent study confirmed that the discrimination in the TIMI score (AUC, 0.867) and GRACE score (AUC, 0.871) shows similarity when predicting in-hospital mortality in STEMI patients [31]. A study used machine learning methods to construct the in-hospital mortality prediction models (with CTREE, Bayes, and generalized linear models) for STEMI patients with data derived from the Chinese Acute Myocardial Infarction Registry. The models achieved relatively higher predictive ability (AUC = 0.80–0.85) [32]. Another independent study applied deep learning-based risk stratification in acute myocardial infarction patients (DAMI) to predict in-hospital mortality rate, and the results showed that the AUC of DAMI was 0.905 in STEMI patients, which significantly outperformed those of the GRACE, acute coronary treatment and intervention outcomes network, and TIMI scores [33]. In addition, a recent study used the SYNTAX score II [34], and another study used Lasso analysis [35], both performed to predict the in-hospital mortality in STEMI patients who underwent pPCI, and the results suggested that the predictability was quite high, and AUC values were 0.927 and 0.990 respectively. In this study, four machine learning models were used. However, an optimal AUC of 0.9273 to predict in-hospital mortality was RAN, which suggested that the prediction accuracy was good. Based on the relevant previous study results, machine learning methods could significantly improve the predictive ability for in-hospital mortality. Figure 6 shows that the Killip class was the most important factor when predicting the in-hospital mortality rate using the RAN model, Alb, CK-MB, stent length, Cr, LVEF, WBC, LDL-C, FMC, hs-CRP, and cTnI. Many studies have proved that Killip class delayed reperfusion, decreased LVEF and Alb, elevated WBC, LDL-C, hs-CRP, CK-MB, and cTnI, increased creatinine, and stent length was associated with STEMI in-hospital mortality [34, 36–38]. More attention should be paid to this, and optimal treatment decisions should be made for STEMI patients with these characteristics.

Some major limitations of this study should be mentioned. First, this study was a retrospective data analysis study, and not every patient had full medical records for clinical data. Therefore, the missing values could not be measured reliably and accurately. Some patients died in the hospital following the pPCI procedure, which meant that some of the relevant data that could have been useful for the study analysis was missing. Inappropriate use of the mean value might lead to deviations in the results. Second, some reference studies reported that BMI was associated with the incidence of NR [39] and in-hospital mortality [11]. However, the combined analysis of BMI was not included in this study. The excessive pursuit of improving D–B time contributed to the loss of information on STEMI patients’ weight and height during admission and transport, which failed to obtain BMI data on all the patients. Third, theoretically, larger sample sizes lead to more robust models, in particular, for machine learning algorithms [40]. This is even more true for neural network algorithms [41]. Therefore, sample-size determination methodologies (SSDMs) are required to estimate the optimum number of participants to arrive at scientifically valid results [42, 43]. Although an attempt was made to collect all the available STEMI patients’ information from four National Chest Pain Centers in the city, the sample size was limited by its restricted geographic scope, and the chest pain center database had not been established for very long. Neural network-based methods performed worse in this study. This was probably because the positive sample size was too small for low statistical power in NNET. In addition, this study was not externally verified in a separate cohort; independent external validation is required to confirm the findings. Fourth, several studies have been carried out on NR and in-hospital mortality prediction [35, 44–47]. They had similar or even better predictions by using generalized linear models or simpler statistical methods than the machine learning approach in this study. Many uncertain factors could influence the occurrence of NR and in-hospital mortality in STEMI patients. In addition to these features used in the models in this study, other potential features are associated with NR and in-hospital mortality, which could be considered predictive risk markers. Future research should focus on designs that include more extensive influencing factors to improve prediction performance. Admission ECG parameters are very important in predicting no-reflow; however, these parameters were not included in this analysis. Therefore, further exploration of how to determine the perfect admission electrocardiographic parameter to predict no-reflow is required. New techniques are required that could contribute to improving the model. For example, a factor disentangling sequential autoencoder approach could help the further intensive analyses and obtain additional information from raw electrocardiograms [48], which could be integrated into the machine learning model; this could be a new direction for future research. Fifth, although this data was obtained from a multicenter, the design was retrospective, and investigating and validating the scoring system was not carried out in the study group; therefore, considering the risk score in patients could provide appropriate therapy for NR patients.

Further, studying the etiopathogenesis of NR at the internal molecular level and investigating the effective strategies for the prevention of NR were absent. Combining machine learning and big data was the optimal aspect of this study; however, the sample sizes were not large enough. More patients from other centers should be included in future machine learning studies.

## Conclusions

The NR phenomenon is a life-threatening challenge for patients with STEMI. It does not have a clear prognosis and associated factors, which might be individual or combined, exhibit the relevant clinical factors. NR is one of the main causes of in-hospital mortality in patients with STEMI. Accurate and interpretable predictions of the incidence of NR and in-hospital mortality are critical in clinical decision-making in STEMI patients. This study used machine learning to establish a predictive model for NR and in-hospital mortality in STEMI patients who underwent pPCI. The results of this study showed that the RAN model that was used to predict NR and in-hospital mortality had high predictive performance and provided significant output features for the prediction of the event, which might help in the early detection and identification and allow the adoption of appropriate clinical interventions to prevent NR and in-hospital deaths, and therefore, improve prognosis and reduce mortality. However, studying the machine learning aspect on a large sample size, combined with a long-term follow-up period, would help to confirm these findings.

## Data Availability

The raw data supporting the conclusions of this article will be made available from the corresponding author on reasonable request.

## Abbreviations

MLA: Machine learning algorithm
NR: No reflow
STEMI: ST-elevation myocardial infarction
pPCI: Primary percutaneous coronary intervention
AUC: Area under the curve
CIs: Confidence intervals
RAN: Random forest algorithm
CTREE: Decision tree
SVM: Support vector machine
NNET: Neural network algorithm
CPCs: Chest pain centers
TIMI: Thrombolysis and thrombin inhibition in myocardial infarction
BMI: Body mass index
cTnI: Cardiac troponin I
CK-MB: Creatine kinase-MB
hs-CRP: High-sensitivity C-reactive protein
WBC: White blood cell
LVEF: Left ventricular ejection fraction
SO-to-FMC: Symptom-onset to-first medical contact
Cr: Creatinine
LDL-C: Low-density lipoprotein cholesterol
RPR: Red cell distribution width–platelet ratio
HDL-C: High-density lipoprotein cholesterol
GRACE: Global Registry of Acute Coronary Events
DAMI: Deep learning-based risk stratification in acute myocardial infarction patients
